# Correction to: Participatory System Dynamics Modeling: Increasing Stakeholder Engagement and Precision to Improve Implementation Planning in Systems

**DOI:** 10.1007/s10488-020-01105-3

**Published:** 2021-01-12

**Authors:** Lindsey Zimmerman, David W. Lounsbury, Craig S. Rosen, Rachel Kimerling, Jodie A. Trafton, Steven E. Lindley

**Affiliations:** 1 National Center for PTSD, Dissemination and Training Division, Veteran Affairs Palo Alto Health Care System, 795 Willow Rd. Bldg. 334 (NC-PTSD), Menlo Park, CA 94025 USA; 2 University of Washington School of Medicine, Seattle, WA USA; 3 Department of Epidemiology and Population Health, Department of Family and Social Medicine, Albert Einstein College of Medicine, Bronx, NY USA; 4 Stanford University School of Medicine, Palo Alto, CA USA; 5 Program Evaluation Resource Center, Center for Innovation To Implementation, Veteran Affairs Palo Alto Health Care System, Menlo Park, CA USA; 6 Veteran Affairs Palo Alto Health Care System, Menlo Park, CA USA

## Correction to: Administration and Policy in Mental Health and Mental Health Services Research (2016) 43:834–849 10.1007/s10488-016-0754-1

The article “Participatory System Dynamics Modeling: Increasing Stakeholder Engagement and Precision to Improve Implementation Planning in Systems”, written by “Lindsey Zimmerman, David W. Lounsbury, Craig S. Rosen, Rachel Kimerling, Jodie A. Trafton, Steven E. Lindley”, was originally published Online First without Open Access. After publication in volume 43, issue 6, pages 834–849 the author decided to opt for Open Choice and to make the article an Open Access publication. Therefore, the copyright of the article has been changed to © The Authors 2020 and the article is forthwith distributed under the terms of the Creative Commons Attribution 4.0 International License, which permits use, sharing, adaptation, distribution and reproduction in any medium or format, as long as you give appropriate credit to the original author(s) and the source, provide a link to the Creative Commons licence, and indicate if changes were made. The images or other third party material in this article are included in the article’s Creative Commons licence, unless indicated otherwise in a credit line to the material. If material is not included in the article’s Creative Commons licence and your intended use is not permitted by statutory regulation or exceeds the permitted use, you will need to obtain permission directly from the copyright holder. To view a copy of this licence, visit http://creativecommons.org/licenses/by/4.0.

The original article has been corrected.

